# Supplementary material for: SENP6-Mediated deSUMOylation of VEGFR2 Enhances Its Cell Membrane Transport in Angiogenesis
Source: Int J Mol Sci. 2023 Jan 29;24(3):2544. doi: 10.3390/ijms24032544 (PMC9916989; doi:10.3390/ijms24032544)
Supplement: Supplementary file 1 [file ijms-24-02544-s001.zip › ijms-2147197-supplementary.pdf]

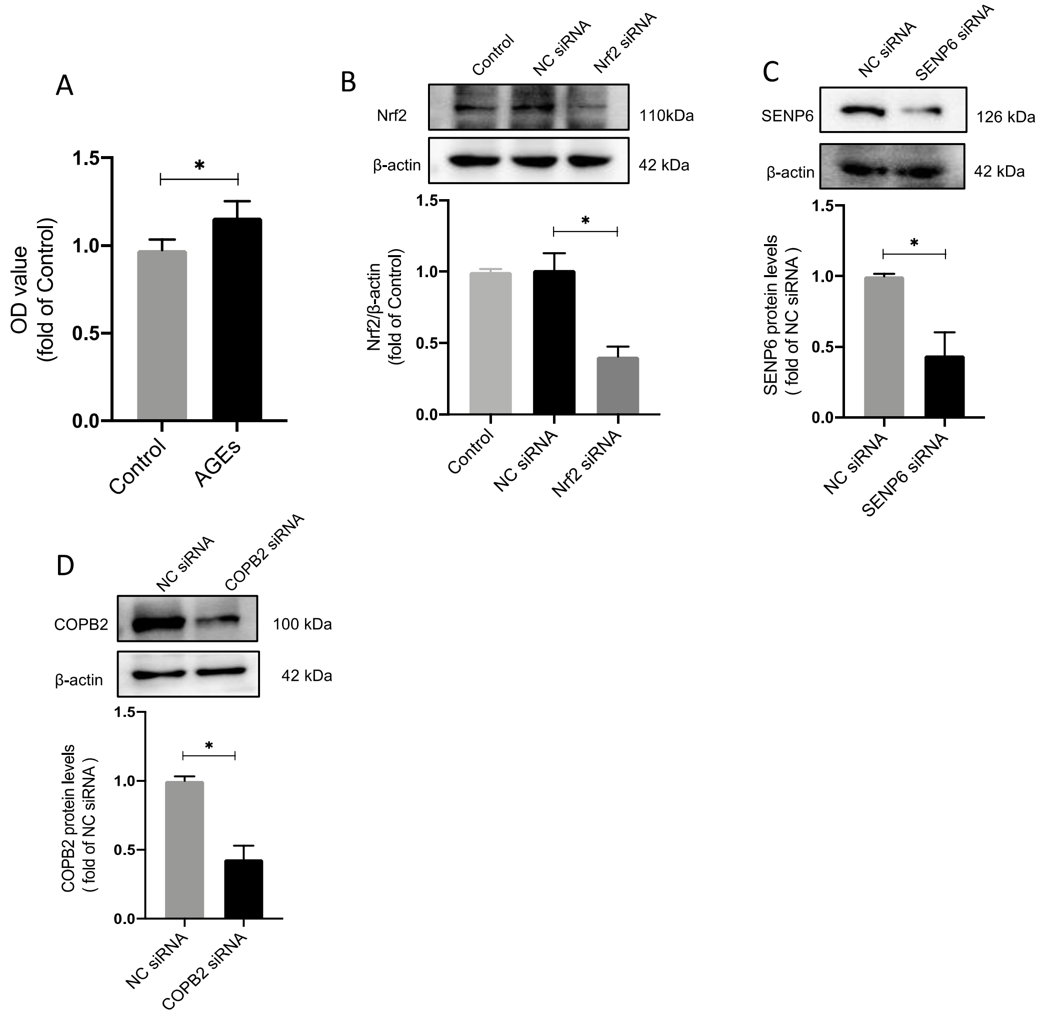

Supplementary Figure S1. (A) cytotoxicity test of AGEs， $n = 3$, ⋆ $p < 0.05$ vs. Control (B) Nrf2 siRNA down-regulated Nrf2 expression. $n = 8$, ⋆ $p < 0.05$ vs. Control (C) SENP6 siRNA down-regulated Nrf2 expression. $n = 5$, ⋆ $p < 0.05$ vs. NC siRNA(D) COPB2 siRNA down-regulated COPB2 expression. $n = 4$, ⋆ $p < 0.05$ vs. NC siRNA. * indicates significance between the indicated groups.
