# Supplementary material for: SENP6-Mediated deSUMOylation of VEGFR2 Enhances Its Cell Membrane Transport in Angiogenesis
Source: Int J Mol Sci. 2023 Jan 29;24(3):2544. doi: 10.3390/ijms24032544 (PMC9916989; doi:10.3390/ijms24032544)

Fig.1. A. Nrf2 (CN)

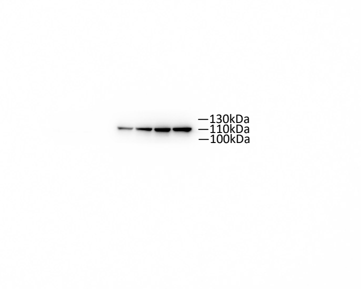

Fig.1. A. LaminB

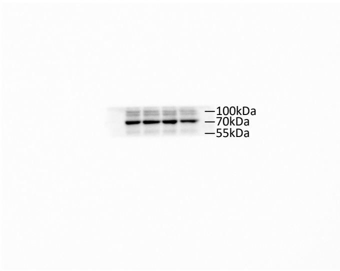

Fig.1. A. Nrf2 (CM)

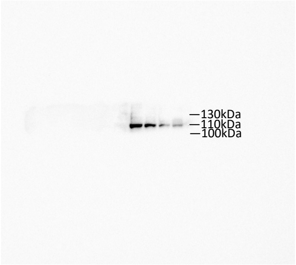

Fig.1. A. β-actin

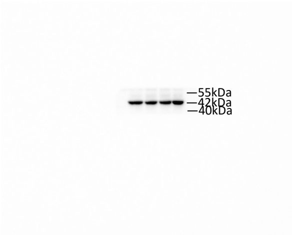

Fig.1. F. VEGFR2

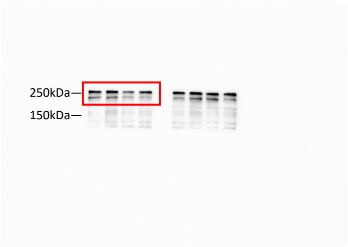

Fig.1. F. β-actin

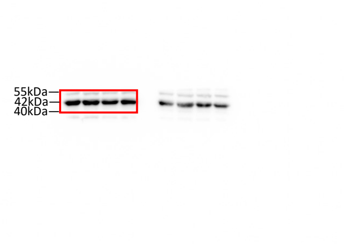

Fig.1. H. VEGFR2

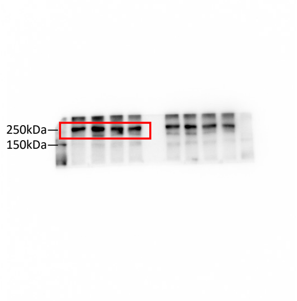

Fig.1. H. β-actin

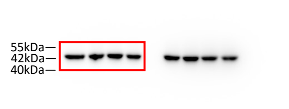

Fig.3. C. SENP6

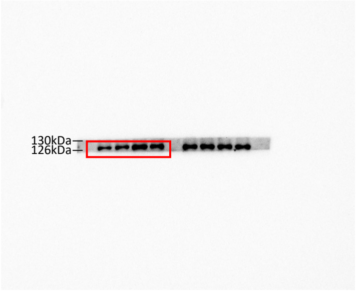

Fig.3. C. β-actin

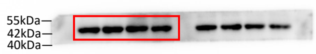

Fig.4. A. VEGFR2

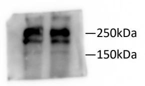

Fig.4. A. β-actin

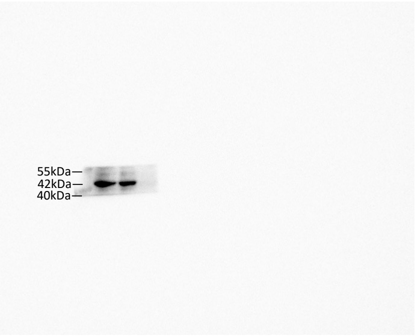

Fig.4. C. SUMO2/3

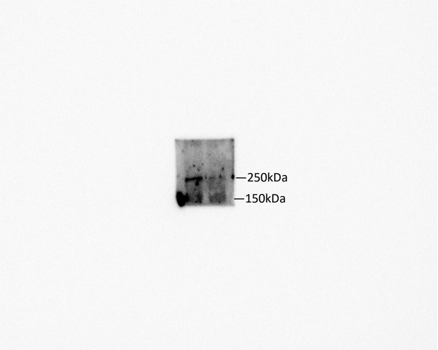

Fig.4. C. VEGFR2

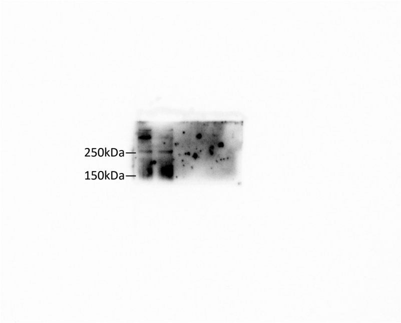

Fig.5. A. VEGFR2

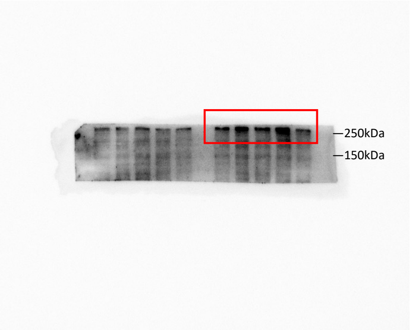

Fig.5. A. β-actin

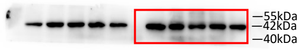

Fig.5. C. VEGFR2

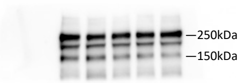

Fig.5. C. β-actin

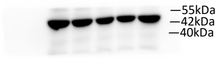

Fig.5. E. VEGFR2

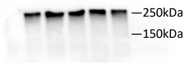

Fig.5. E. ATP1A1

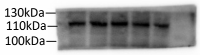

Fig.5. I. COPB2

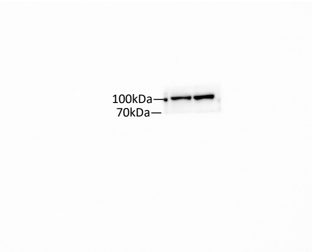

Fig.5. I. β-actin

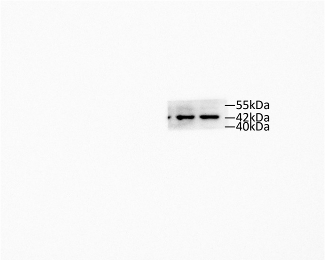

Fig.5. L. VEGFR2

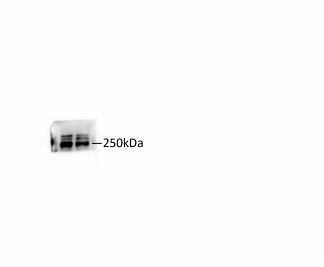

Fig.5. L. ATP1A1

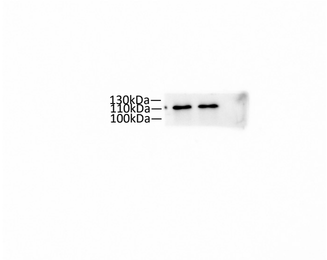

Fig.6. A. VEGF

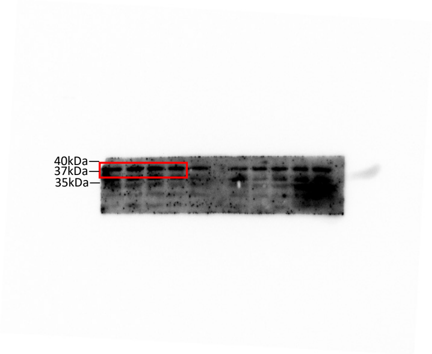

Fig.6. A. β-actin

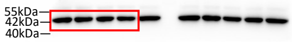

Fig.6. C. VEGF

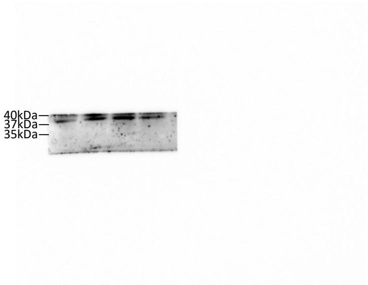

Fig.6. C. β-actin

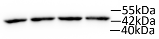

Supplemental Fig. S1, A. Nrf2

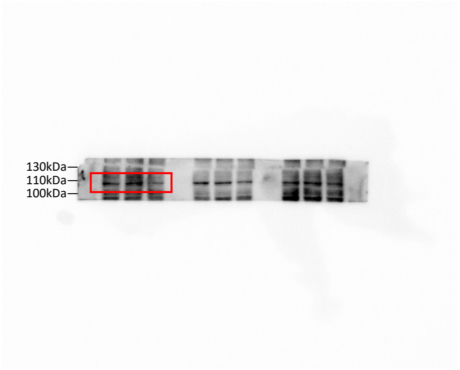

Supplemental Fig. S1, A. β-actin

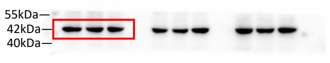

Supplemental Fig. S1, B. SENP6

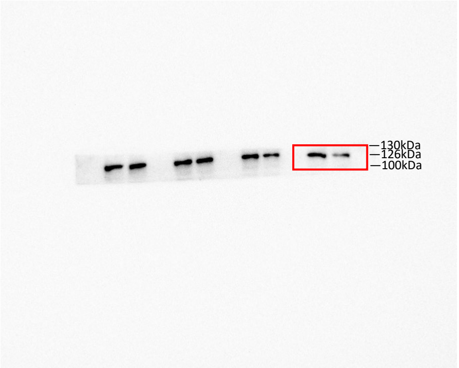

Supplemental Fig. S1, B. β-actin

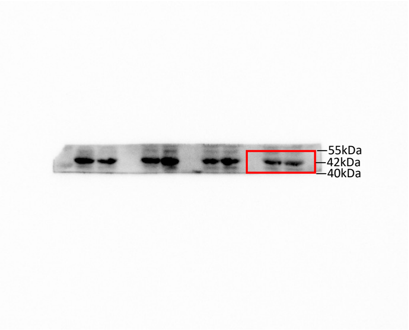

Supplemental Fig. S1, C. COPB2

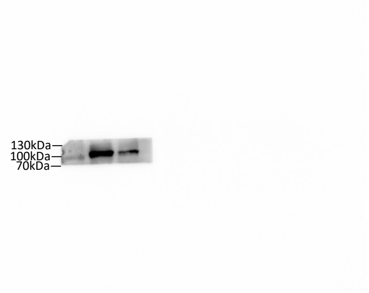

Supplemental Fig. S1, C. β-actin

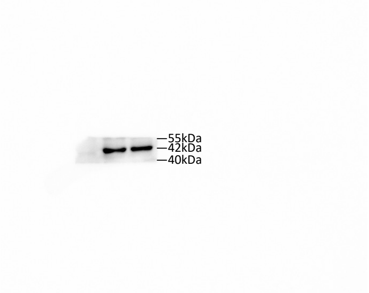

Supplement: Supplementary file 1 [file ijms-24-02544-s001.zip › Original Images .pdf]
